# Evaluation of Concomitant Systemic Treatment in Older Adults With Head and Neck Squamous Cell Carcinoma Undergoing Definitive Radiotherapy

**DOI:** 10.1001/jamanetworkopen.2023.0090

**Published:** 2023-02-20

**Authors:** Alexander Rühle, Sebastian Marschner, Marlen Haderlein, Alexander Fabian, Maria Weymann, Max Behrens, Carolin Senger, Daniel R. Dickstein, Johannes Kraft, Jens von der Grün, Eric Chen, Todd Aquino-Michaels, Justus Domschikowski, Amanda Bickel, Alev Altay-Langguth, Goda Kalinauskaite, Victor Lewitzki, Konstantinos Ferentinos, Constantinos Zamboglou, Sören Schnellhardt, Erik Haehl, Simon K.B. Spohn, Eleni Gkika, Daniela Zöller, Matthias Guckenberger, Volker Budach, Claus Belka, Richard Bakst, Arnulf Mayer, Heinz Schmidberger, Anca-Ligia Grosu, Panagiotis Balermpas, Carmen Stromberger, Nils H. Nicolay

**Affiliations:** 1 Department of Radiation Oncology, University of Freiburg–Medical Center, Freiburg, Germany; 2 German Cancer Consortium (DKTK) Partner Site Freiburg, German Cancer Research Center (DKFZ), Heidelberg, Germany; 3 Department of Radiation Oncology, University Hospital, LMU Munich, Munich, Germany; 4 DKTK Partner Site Munich, German Cancer Research Center, Heidelberg, Germany; 5 Department of Radiation Oncology, Universitätsklinikum Erlangen, Friedrich-Alexander-Universität Erlangen-Nürnberg, Erlangen, Germany; 6 Comprehensive Cancer Center Erlangen-EMN, Universitätsklinikum Erlangen, Friedrich-Alexander-Universität Erlangen-Nürnberg, Erlangen, Germany; 7 Department of Radiation Oncology, University Hospital Schleswig-Holstein, Campus Kiel, Kiel, Germany; 8 Institute of Medical Biometry and Statistics, Faculty of Medicine and Medical Center-University of Freiburg, Freiburg, Germany; 9 Department of Radiation Oncology, Charité–Universitätsmedizin Berlin, corporate member of Freie Universität Berlin, Humboldt-Universität zu Berlin, and Berlin Institute of Health, Germany, Berlin, Germany; 10 DKTK Partner Site Berlin, DKFZ, Neuenheimer Feld 280, Heidelberg, Germany; 11 Department of Radiation Oncology, Icahn School of Medicine at Mount Sinai, New York, New York; 12 Department of Radiation Oncology, University Hospital Würzburg, Würzburg, Germany; 13 Department of Radiotherapy and Oncology, Goethe University Frankfurt, Frankfurt am Main, Germany; 14 DKTK Partner Site Frankfurt, German Cancer Research Center, Heidelberg, Germany; 15 Department of Radiation Oncology, University Hospital Zurich, University of Zurich, Zurich, Switzerland; 16 Department of Radiation Oncology, University Hospitals Seidman Cancer Center, Cleveland, Ohio; 17 Department of Radiation Oncology, German Oncology Center, European University of Cyprus, Limassol, Cyprus; 18 Department of Radiation Oncology and Radiation Therapy, University Medical Center Mainz, Mainz, Germany; 19 DKTK Partner Site Mainz, German Cancer DKFZ, Heidelberg, Germany; 20 Department of Radiation Oncology, University of Leipzig, Leipzig, Germany; 21 Comprehensive Cancer Center Central Germany, Partner Site Leipzig, Leipzig, Germany

## Abstract

**Importance:**

The number of older adults with head and neck squamous cell carcinoma (HNSCC) is increasing, and these patients are underrepresented in clinical trials. It is unclear whether the addition of chemotherapy or cetuximab to radiotherapy is associated with improved survival in older adults with HNSCC.

**Objective:**

To examine whether the addition of chemotherapy or cetuximab to definitive radiotherapy is associated with improved survival in patients with locoregionally advanced (LA) HNSCC.

**Design, Setting, and Participants:**

The Special Care Patterns for Elderly HNSCC Patients Undergoing Radiotherapy (SENIOR) study is an international, multicenter cohort study including older adults (≥65 years) with LA-HNSCCs of the oral cavity, oropharynx/hypopharynx, or larynx treated with definitive radiotherapy, either alone or with concomitant systemic treatment, between January 2005 and December 2019 at 12 academic centers in the US and Europe. Data analysis was conducted from June 4 to August 10, 2022.

**Interventions:**

All patients underwent definitive radiotherapy alone or with concomitant systemic treatment.

**Main Outcomes and Measures:**

The primary outcome was overall survival. Secondary outcomes included progression-free survival and locoregional failure rate.

**Results:**

Among the 1044 patients (734 men [70.3%]; median [IQR] age, 73 [69-78] years) included in this study, 234 patients (22.4%) were treated with radiotherapy alone and 810 patients (77.6%) received concomitant systemic treatment with chemotherapy (677 [64.8%]) or cetuximab (133 [12.7%]). Using inverse probability weighting to attribute for selection bias, chemoradiation was associated with longer overall survival than radiotherapy alone (hazard ratio [HR], 0.61; 95% CI, 0.48-0.77; *P* < .001), whereas cetuximab-based bioradiotherapy was not (HR, 0.94; 95% CI, 0.70-1.27; *P* = .70). Progression-free survival was also longer after the addition of chemotherapy (HR, 0.65; 95% CI, 0.52-0.81; *P* < .001), while the locoregional failure rate was not significantly different (subhazard ratio, 0.62; 95% CI, 0.30-1.26; *P* = .19). The survival benefit of the chemoradiation group was present in patients up to age 80 years (65-69 years: HR, 0.52; 95% CI, 0.33-0.82; 70-79 years: HR, 0.60; 95% CI, 0.43-0.85), but was absent in patients aged 80 years or older (HR, 0.89; 95% CI, 0.56-1.41).

**Conclusions and Relevance:**

In this cohort study of older adults with LA- HNSCC, chemoradiation, but not cetuximab-based bioradiotherapy, was associated with longer survival compared with radiotherapy alone.

## Key Points

**Question:**

Is the addition of concomitant systemic treatment to definitive radiotherapy associated with longer survival in older patients with locoregionally advanced head and neck squamous cell carcinoma?

**Findings:**

In this cohort study of 1044 older adults (aged ≥65 years) with head and neck squamous cell carcinoma, overall survival and progression-free survival were longer in the chemoradiation than in the radiotherapy group, even after adjusting for potential confounders. In contrast, the addition of cetuximab was not associated with improved survival or locoregional control.

**Meaning:**

The results of this analysis suggest that older patients with locoregionally advanced head and neck squamous cell carcinoma may benefit from the addition of chemotherapy, but not cetuximab.

## Teaser

This cohort study examines the outcomes of adding chemotherapy or cetuximab to radiotherapy treatment in older adults with locoregionally advanced head and neck squamous cell carcinoma.

## Introduction

Head and neck squamous cell carcinoma (HNSCC) is the sixth most common cancer worldwide and results in approximately 450 000 deaths per year.^1^ Following the demographic change, the proportion of older patients with HNSCCs is continuously increasing.^2^

The landmark Meta-analysis of Chemotherapy in Head and Neck Cancer (MACH-NC) study regarding the role of concomitant chemotherapy in head and neck cancer detected a significant survival benefit for the addition of chemotherapy to definitive radiotherapy in patients with nonmetastatic HNSCC; however, the benefit was found to be decreasing with higher patient age and to be lost in patients aged 70 years or older.^3^ Although the epidermal growth factor receptor inhibitor cetuximab is considered a treatment alternative for patients with HNSCC not eligible for cisplatin therapy, the subgroup analysis of the key phase 3 study could not detect a benefit for the addition of cetuximab in older (≥65 years) patients either.^4^ Therefore, based on the available data, there is no clear role for the addition of systemic therapy to radiotherapy in older patients with HNSCCs.

However, the included trials of the MACH-NC study (time span 1965-2012) as well as the Bonner et al^4^ trial were conducted in an era in which modern radiotherapy treatment techniques, such as intensity-modulated radiotherapy and modern supportive treatments, were not widely available. In addition, the proportion of older patients was limited in these studies, making meaningful statistical analyses for this subgroup difficult. Furthermore, human papillomavirus (HPV) analyses were missing in the MACH-NC study, complicating the extrapolation of these data to older patients with HPV-related oropharyngeal cancers, whose numbers have continuously increased since then and who commonly exhibit better performance and fewer comorbidities than patients with HPV-negative HNSCCs.^5,6,7^

Previously published larger database analyses, which included patients treated between 1992 and 2007 in the Surveillance, Epidemiology, and End Results (SEER) registry and between 1998 and 2011 in the National Cancer Database (NCDB), predominantly included patients treated before use of intensity-modulated radiotherapy and provided no information regarding locoregional control.^8,9^ Due to differences in the patient population and the statistical methods, the SEER and NCDB analyses reported contradictory findings. While there was a survival benefit noted with the addition of chemotherapy in patients aged between 71 and 81 years and with low comorbidity burden and either T1-2 N2-3 or T3-4 N0-3 disease in the NCDB analysis, patients treated with chemoradiation had reduced survival compared with radiotherapy even after propensity score matching according to the SEER analysis. Another NCDB-based analysis observed an association between superior survival and the addition of concomitant chemotherapy to radiotherapy in older patients with locoregionally advanced (LA) oropharyngeal carcinoma, irrespective of the tumoral HPV status.^10^

Considering these discrepancies and the limitations of these older analyses, we analyzed the value of concomitant systemic treatment during radiotherapy within a large, contemporary multicenter cohort of older adults with LA-HNSCCs treated with state-of-the-art techniques regarding radiotherapy, chemotherapy, and supportive treatments. In addition to the association between treatment type and overall survival (OS) or progression-free survival (PFS), we aimed to examine the association between concomitant systemic treatment and locoregional failure (LRF) and distant metastasis (DM) rate. To our knowledge, the present international multicenter study is the largest analysis regarding the value of concomitant systemic treatment for older patients with LA-HNSCCs in which PFS, LRF, and DM rates were analyzed in addition to OS and in which individual patient data, including potential confounding variables, such as comorbidities, smoking history, and HPV status, were available.

## Methods

### Study Design and Patient Population

An international cohort study (Special Care Patterns for Elderly HNSCC Patients Undergoing Radiotherapy [SENIOR], NCT05337631) with individual patient and treatment data of older patients with HNSCC was created in order to analyze the value of concomitant systemic treatment to definitive radiotherapy. This study followed the Strengthening the Reporting of Observational Studies in Epidemiology (STROBE) reporting guideline for cohort studies. Patients with LA-HNSCCs of the oral cavity, oropharynx/hypopharynx, or larynx undergoing definitive radiotherapy, alone or with simultaneous systemic treatment, between January 2005 and December 2019 were retrospectively analyzed. Deidentified data were obtained from 12 academic centers in the US, Germany, Switzerland, and Cyprus. The ethics committee of the University of Freiburg, Germany, approved this study in general, and the institutional review boards at each participating center approved data collection and data sharing with the responsible study center.

Older patients (≥65 years) who were diagnosed with LA (cT3-4 and/or cN+) HNSCCs of the oral cavity, oropharynx, hypopharynx, or larynx between 2005 and 2019 and received definitive photon radiotherapy, as a single modality or with concomitant systemic treatment, were included in this analysis. Exclusion criteria were induction or adjuvant chemotherapy, history of head and neck carcinomas or radiotherapy in the head and neck region, DMs at the initiation of treatment, or LA-HNSCCs of the nasopharynx, salivary glands, skin, or cancers of an unknown primary.

The *American Joint Committee on Cancer Cancer Staging Manual Seventh Edition* staging was applied for all patients.^11^ The Charlson Comorbidity Index was calculated as reported in the literature^12^ (without incorporating patient age) with the difference that the primary tumor itself was not included in the calculation.

### Statistical Analysis

For this analysis, conducted from June 4 to August 10, 2022, the SENIOR database entry was closed on June 3, 2022. Death, local or locoregional progression, and development of DMs were events regarding PFS. All end points were calculated from the start of radiotherapy until death, the respective event, or last follow-up date. Patients were right-censored at the last date of follow-up. Multiple imputation of missing data (eTable 1 in Supplement 1) was conducted using k-nearest neighbor imputation, in which the 5 nearest neighbors were computed using a variation of the Gower distance.^13^ Herein, the median value was used to impute numerical missing data and the most common category was used to impute categorical missing data. In addition to the imputation-based cohort analysis, a complete case analysis was conducted. Inverse probability weighting was used to balance the groups regarding the baseline covariates. Individual inverse probability weights were stabilized in order to improve the estimate of the variance of the treatment effect and were pruned to the 99th percentile to avoid severe outliers.^14,15^ Standardized mean differences were examined to check the balance of the baseline covariates after IPW (eFigure 1 in Supplement 1). For a standardized mean difference less than 0.2, an adequate balance of the covariate was assumed.^16^

The OS and PFS were calculated using the Kaplan-Meier method, and log-rank tests were used to compare OS and PFS between treatment groups. In the IPW-adjusted treatment groups, weighted Kaplan-Meier curves and weighted log-rank tests were used accordingly. Furthermore, the restricted mean survival time difference at 24 months after the start of treatment was determined for the different treatment groups.^17^ Both the incidence of LRFs and the incidence of DMs were evaluated using proportional hazards models for the subdistribution of LRFs or DMs, with death as competing risk.^18^ Multivariate Cox proportional hazards analyses of both the unadjusted and IPW-adjusted groups were conducted. In an IPW-adjusted subgroup analysis, a weighted Cox proportional hazards regression model was estimated within each level of each covariate.

Statistical analyses were conducted using R, version 4.1.3 (R Foundation for Statistical Computing). All analyses were exploratory. As a result, *P* values and 95% CIs were not corrected for multiple comparisons. Findings were considered significant at 2-sided *P* values <.05.

## Results

### Characteristics of the Study Cohort

A total of 1044 patients (734 [70.3%] men, 310 [29.7%] women) receiving definitive radiotherapy (Table 1; eTable 2 in Supplement 1), either as a single modality (234 [22.4%]) or with concomitant systemic treatment (810 [77.6%]), were included in this analysis. Median age at radiotherapy initiation was 73 (IQR, 69-78) years, with 286 patients (27.4%) aged 65 to 69 years, 571 (54.7%) aged 70 to 79 years, and 187 (17.9%) aged 80 years or older. The median Eastern Cooperative Oncology Group (ECOG) performance status (range, 0-4 with highest number indicating worst performance status) was 1 (IQR, 0-1), while the median Charlson Comorbidity Index (range, 0-11 with highest number indicating worst comorbidity burden) was 2 (IQR, 0-3) points. Among the 304 oropharyngeal carcinomas with known HPV status, 186 (61.2%) were HPV-positive. Most patients (863 [82.7%]) had T3-T4 tumors and exhibited locoregional nodal metastases (847 [81.1%]). The median radiotherapy dose was 70.0 Gy (IQR, 69.3-70.0 Gy), and 933 patients (89.4%) completed the radiotherapy course with the prescribed radiation dose.

**Table 1.** Baseline Characteristics of Patients Aged 65 Years and Older Who Underwent Definitive Radiotherapy for Locally Advanced Head and Neck Squamous Cell Carcinoma

Characteristic	No. (%) of patients		
	Radiotherapy (n = 234)	CRT (n = 677)	Radiotherapy with cetuximab (n = 133)
Age, median (IQR), y	79 (74-83)	71 (68-76)	74 (70-79)
Sex			
Female	75 (32.1)	211 (31.2)	24 (18.0)
Male	159 (67.9)	466 (68.8)	109 (82.0)
ECOG status			
0	38 (16.2)	210 (31.0)	21 (15.8)
1	106 (45.3)	354 (52.3)	70 (52.6)
≥2	85 (36.3)	102 (15.1)	37 (27.8)
Missing	5 (2.1)	11 (1.6)	5 (3.8)
CCI, median (IQR)^a^	2 (1-3)	1 (0-3)	2 (1-3)
Smoking			
Never/limited	82 (35.0)	181 (26.7)	43 (32.3)
Smoking >10 pack-years	123 (52.6)	380 (56.1)	69 (51.9)
Missing	29 (12.4)	116 (17.1)	21 (15.8)
Localization			
Oral cavity	54 (23.1)	93 (13.7)	13 (9.8)
Oropharynx	99 (42.3)	356 (52.6)	68 (51.1)
Hypopharynx	35 (15.0)	112 (16.5)	24 (18.0)
Larynx	41 (17.5)	86 (12.7)	25 (18.8)
Oropharynx/hypopharynx	5 (2.1)	30 (4.4)	3 (2.3)
Clinical T category			
cT1	15 (6.4)	28 (4.1)	3 (2.3)
cT2	30 (12.8)	81 (12.0)	24 (18.0)
cT3	76 (32.5)	220 (32.5)	48 (36.1)
cT4	113 (48.3)	348 (51.4)	58 (43.6)
Clinical N category			
cN0	57 (24.4)	115 (17.0)	24 (18.0)
cN1	37 (15.8)	84 (12.4)	15 (11.3)
cN2a	8 (3.4)	14 (2.1)	2 (1.5)
cN2b	59 (25.2)	145 (21.4)	30 (22.6)
cN2c	23 (9.8)	141 (20.8)	20 (15.0)
cN2, not specified	31 (13.2)	140 (20.7)	32 (24.1)
cN3	18 (7.7)	38 (5.6)	10 (7.5)
Missing	1 (0.4)	0	0
HPV status of oropharynx carcinomas^b^			
HPV-positive	39 (39.4)	122 (34.3)	25 (36.8)
HPV-negative	26 (26.3)	76 (21.3)	16 (23.5)
Missing	34 (34.3)	158 (44.4)	27 (39.7)
Radiotherapy dose, median (IQR), Gy	69.3 (60.0-70.0)	70.0 (69.3-70.4)	70.0 (70.0-70.0)
Radiotherapy completion			
Completed	202 (86.3)	611 (90.3)	120 (90.2)
Not completed	32 (13.7)	66 (9.7)	13 (9.8)

Abbreviations: CCI, Charlson Comorbidity Index (range, 0-11 with highest number indicating worst comorbidity burden); CRT, chemoradiation; ECOG, Eastern Cooperative Oncology Group (range, 0-4 with highest number indicating worst performance status); HPV, human papillomavirus.

^a^ Determined for 1042 patients.

^b^ For radiotherapy, n = 99; for CRT, n = 356, and for radiotherapy with cetuximab, n = 68.

A total of 810 patients (77.6%) received concomitant systemic treatment, either chemotherapy (677 [64.8%]) or cetuximab (133 [12.7%]). Among the patients receiving chemoradiation, cisplatin (298 [44.0%]), cisplatin plus fluorouracil (137 [20.2%]), and carboplatin (68 [10.0%]) were the most common regimens (eTable 3 in Supplement 1). The median cumulative cisplatin dose in patients treated with cisplatin monotherapy was 175 mg/m^2^ (IQR, 120-200 mg/m^2^). Of the 298 patients who received concomitant cisplatin, 147 (49.3%) were treated with cisplatin, 30 to 40 mg/m^2^, weekly; 8 (2.7%) with cisplatin, 100 mg/m^2^, on days 1, 22, and 43; and the remaining patients with other fractionated cisplatin regimens (eg, cisplatin 15 to 20 mg/m^2^, on days 1 to 5 and days 29 to 33, or cisplatin, 20 mg/m^2^, on days 1 to 5, days 22 to 26, and days 43 to 47).

### Association of Concomitant Systemic Treatment With OS

The median follow-up time amounted to 56 months (95% CI, 50-62 months). At the time of analysis, 569 deaths (54.5%), 246 LRFs (23.6%), and 133 DMs (12.7%) had occurred. There were 85 deaths (8.1%) within the first 2 months after the start of radiotherapy. Median OS was 36 months (95% CI, 33-43 months) and median PFS was 20 months (95% CI, 17-26 months). The 2-year incidence of LRFs was 22.5% (95% CI, 19.9%-25.2%).

**Figure 1.**
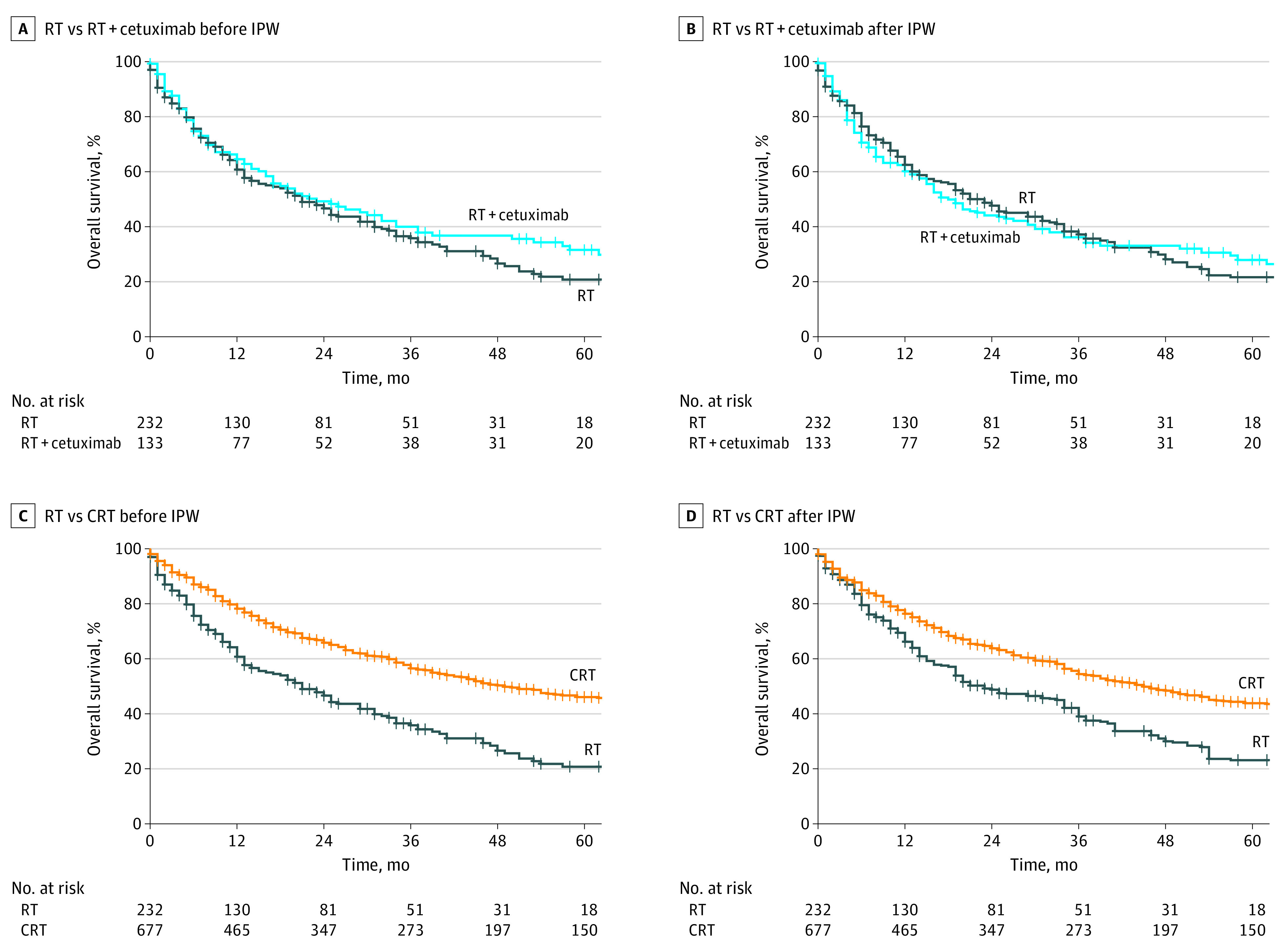
Overall Survival of Older Patients (≥65 Years) With Head and Neck Squamous Cell Carcinoma Receiving Radiotherapy (RT) Alone or With Concomitant Systemic Treatment A, RT vs RT with cetuximab before inverse probability weighting (IPW); hazard ratio (HR), 0.81 (95% CI, 0.62-1.07); *P* = .13. B, RT vs RT with cetuximab after IPW; HR, 0.94 (95% CI, 0.70-1.27); *P* = .70. C, RT vs chemoradiation (CRT) before IPW; HR, 0.52 (95% CI, 0.43-0.63); *P* < .001. D, RT vs CRT after IPW; HR, 0.61 (95% CI, 0.48-0.77); *P* < .001. Inverse probability weighting was used to balance the groups regarding the baseline covariates including age, sex, Eastern Cooperative Oncology Group (ECOG) status, comorbidities, smoking, tumor localization, T category, N category, and human papillomavirus status. Note that the number of patients in the RT group is lower than in the overall population (232 instead of 234), as 2 patients with ECOG status 4 could not be matched using IPW.

In the unadjusted cohort, the addition of chemotherapy to definitive radiotherapy was associated with significantly improved OS compared with radiotherapy alone (hazard ratio [HR], 0.52; 95% CI, 0.43-0.63; *P* < .001) (Figure 1C). Application of concomitant chemotherapy resulted in an improved median OS (49 months; 95% CI, 41-63 months) compared with radiotherapy alone (21 months; 95% CI, 15-29 months; *P* < .001; log-rank test). The restricted mean survival time difference after 24 months amounted to 3.4 months (95% CI, 2.0-4.7 months; *P* < .001) in favor of the chemoradiation group. In contrast, the addition of cetuximab to radiotherapy did not increase OS when compared with radiotherapy alone (HR, 0.81; 95% CI, 0.62-1.07; *P* = .13) (Figure 1A), and there was no significantly restricted mean survival time difference after 24 months between these groups (0.4 months; 95% CI, −1.6 to 2.4 months; *P* = .68).

In the Kaplan-Meier analysis of the IPW-adjusted cohorts, patients who were treated with chemoradiation still exhibited longer OS than patients undergoing radiotherapy alone (HR, 0.61; 95% CI, 0.48-0.77; *P* < .001) (Figure 1D). Median OS was prolonged from 23 months (95% CI, 16-37 months) in the radiotherapy group to 45 months (95% CI, 37-55 months; *P* < .001; log-rank test) in the chemoradiation group. The 24-month restricted mean survival time difference was 2.1 months (95% CI, 2.1-2.2 months; *P* < .001) after IPW, favoring the chemoradiation group. In contrast, the addition of cetuximab to radiotherapy was not associated with longer OS in older patients with LA-HNSCC (HR, 0.94; 95% CI, 0.70-1.27; *P* = .70; median OS, 23 vs 18 months; *P* = .70; log-rank test) (Figure 1B). The addition of chemotherapy was associated with improved OS and PFS also in the complete case analysis (eFigure 2, eFigure 3 in Supplement 1).

In the multivariate Cox proportional regression analysis, the addition of chemotherapy to radiotherapy was associated with a reduced hazard of death both in the unadjusted (HR, 0.62; 95% CI, 0.49-0.78; *P* < .001) and IPW-adjusted (HR, 0.59; 95% CI, 0.46-0.76; *P* < .001) cohorts (Table 2; eTable 4 in Supplement 1). Adding cetuximab to radiotherapy was not associated with a reduced hazard of death in the multivariate analysis, in either the unadjusted (HR, 0.90; 95% CI, 0.67-1.22; *P* = .51) or the IPW-adjusted (HR, 0.93; 95% CI, 0.68-1.28; *P* = .66) analysis.

**Table 2.** Cox Proportional Hazards Regression Analysis for Overall Survival in Patients Aged 65 Years and Older Who Were Treated With Definitive Radiotherapy for Locally Advanced Head and Neck Squamous Cell Carcinoma^a^

Characteristic	HR (95% CI)	*P* value
**Radiotherapy vs chemoradiation**		
Age	1.03 (1.01-1.05)	.005
Sex (reference, female)	1.29 (1.02-1.64)	.03
ECOG status (reference, 0)		
1	1.57 (1.20-2.05)	.001
2	1.88 (1.33-2.67)	<.001
3	1.62 (0.73-3.58)	.24
CCI	1.04 (0.98-1.10)	.22
Smoking (reference, never/limited smoking)	1.20 (0.93-1.55)	.17
Localization (reference, oral cavity)		
Oropharynx	0.54 (0.40-0.73)	<.001
Hypopharynx	0.62 (0.46-0.84)	.002
Larynx	0.50 (0.34-0.73)	<.001
Oropharynx/hypopharynx (multilevel)	0.51 (0.29-0.90)	.02
Clinical T category (reference, cT1)		
cT2	0.98 (0.56-1.71)	.95
cT3	1.23 (0.76-1.98)	.41
cT4	1.53 (0.95-2.45)	.08
Clinical N category (reference, cN0)		
cN1	1.15 (0.78-1.70)	.47
cN2	1.58 (1.17-2.13)	.003
cN3	2.14 (1.36-3.34)	<.001
HPV status (reference, HPV-positive)	2.22 (1.47-3.33)	<.001
Concomitant chemotherapy	0.59 (0.46-0.76)	<.001
**Radiotherapy vs radiotherapy plus cetuximab**		
Age	1.02 (1.00-1.04)	.07
Sex (reference, female)	0.94 (0.66-1.32)	.71
ECOG status (reference, 0)		
1	1.33 (0.88-2.00)	.17
2	1.81 (1.10-2.95)	.02
3	2.18 (1.02-4.63)	.04
CCI	1.03 (0.95-1.12)	.42
Smoking (reference, never/limited smoking)	1.60 (1.16-2.20)	.004
Localization (reference, oral cavity)		
Oropharynx	0.44 (0.28-0.69)	<.001
Hypopharynx	0.72 (0.44-1.19)	.20
Larynx	0.48 (0.29-0.78)	.003
Oropharynx/hypopharynx (multilevel)	0.34 (0.09-1.20)	.09
Clinical T category (reference, cT1)		
cT2	1.64 (0.75-3.57)	.21
cT3	2.11 (1.03-4.34)	.04
cT4	2.38 (1.15-4.93)	.02
Clinical N category (reference, cN0)		
cN1	0.77 (0.47-1.24)	.28
cN2	1.20 (0.82-1.76)	.35
cN3	1.51 (0.85-2.69)	.16
HPV status (reference, HPV-positive)	1.61 (0.97-2.63)	.06
Concomitant cetuximab	0.93 (0.68-1.28)	.66

Abbreviations: CCI, Charlson Comorbidity Index (range, 0-11 with highest number indicating worst comorbidity burden); ECOG, Eastern Cooperative Oncology Group (range, 0-4 with highest number indicating worst performance status); HPV, human papillomavirus; HR, hazard ratio; IPW, inverse probability weighting.

^a^ Inverse probability weighting was performed to control for imbalances among the baseline variables; herein, IPW was performed separately for each comparison (radiotherapy vs chemoradiation, radiotherapy vs radiotherapy with cetuximab).

### Association of Concomitant Systemic Treatment With PFS and Incidence of LRFs and DMs

**Figure 2.**
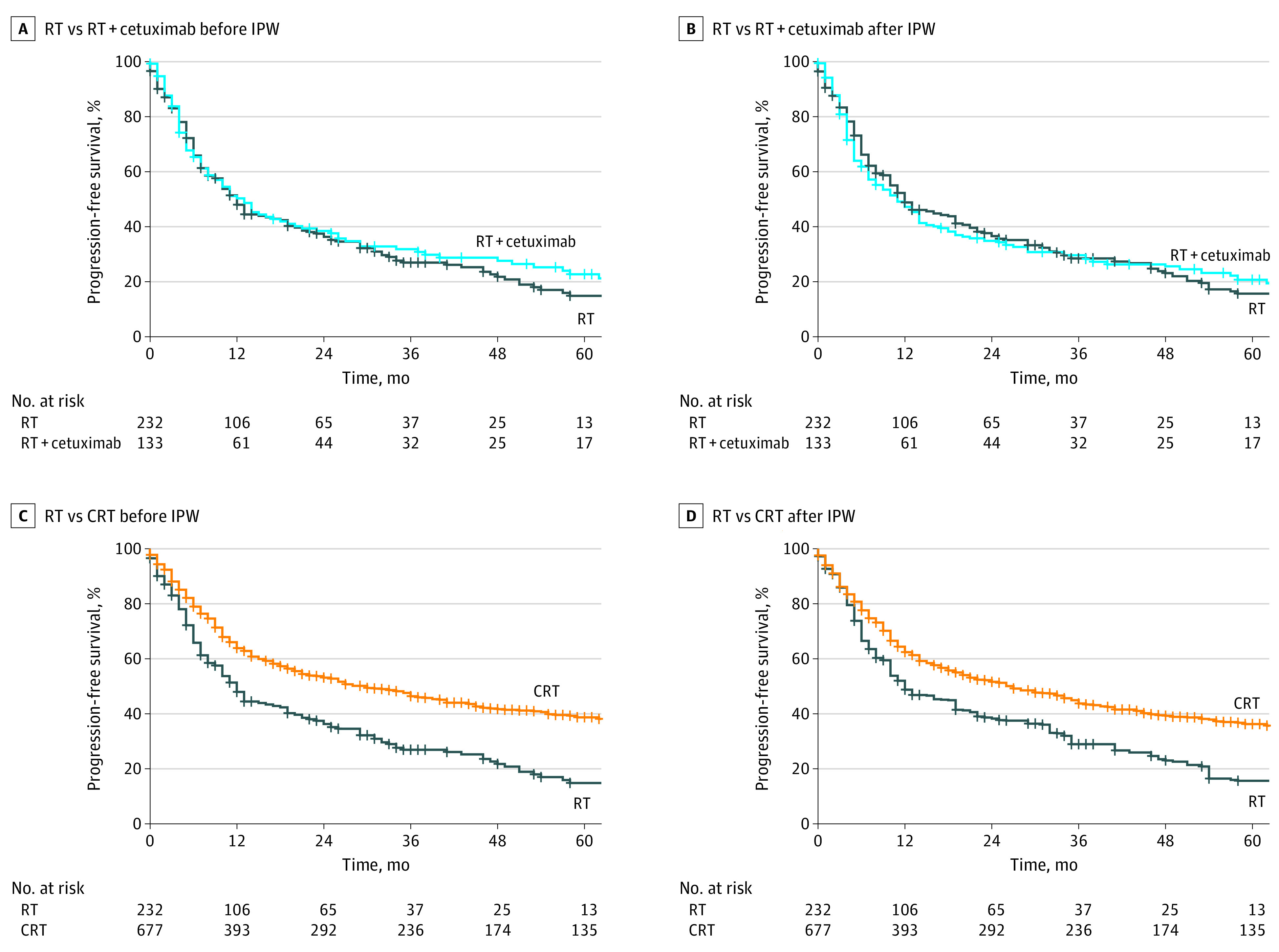
Progression-Free Survival of Older Patients (≥65 Years) With Head and Neck Squamous Cell Carcinoma Receiving Radiotherapy (RT) Depending on Concomitant Systemic Treatment A, RT vs RT with cetuximab before inverse probability weighting (IPW); hazard ratio (HR), 0.88 (95% CI, 0.68-1.13); *P* = .32. B, RT vs RT with cetuximab after IPW; HR, 0.98 (95% CI, 0.75-1.29); *P* = .90. C, RT vs chemoradiation (CRT) before IPW; HR, 0.58 (95% CI, 0.48-0.69); *P* < .001. D, RT vs CRT after IPW; HR, 0.65 (95% CI, 0.52-0.81); *P* < .001. Inverse probability weighting was used to balance the groups regarding the baseline covariates including age, sex, Eastern Cooperative Oncology Group (ECOG) status, comorbidities, smoking, tumor localization, T category, N category, and human papillomavirus status. Note that the number of patients in the RT group is lower than in the overall population (232 instead of 234), as 2 patients with ECOG status 4 could not be matched using IPW.

Prior to IPW, PFS was significantly longer in the chemoradiation than in the radiotherapy group (HR, 0.58; 95% CI, 0.48-0.69; *P* < .001) (Figure 2C), whereas there was no significant difference between the cetuximab-based bioradiotherapy and the radiotherapy group (HR, 0.88; 95% CI, 0.68-1.13; *P* = .32) (Figure 2A). After adjustment for several confounding variables by using IPW, patients in the chemoradiation group still exhibited longer PFS than patients in the radiotherapy group (HR, 0.65; 95% CI, 0.52-0.81; *P* < .001) (Figure 2D). The addition of cetuximab to definitive radiotherapy was not associated with PFS according to the IPW-adjusted Kaplan-Meier analyses (HR, 0.98; 95% CI, 0.75-1.29; *P* = .90) (Figure 2B). In the multivariate analysis, chemoradiation (unadjusted cohort: HR, 0.69; 95% CI, 0.56-0.85; *P* < .001; IPW-adjusted cohort: HR, 0.64; 95% CI, 0.51-0.80; *P* < .001) was still associated with a decreased hazard of death or progression, whereas radiotherapy plus cetuximab (unadjusted cohort: HR, 0.99; 95% CI, 0.75-1.31; *P* = .97; IPW-adjusted cohort: HR, 1.00; 95% CI, 0.74-1.34; *P* = .98) was not (eTable 5, eTable 6 in Supplement 1).

The numerical incidence of LRFs was lower in the chemoradiation than in the radiotherapy group, but statistical significance was not reached (subhazard ratio [SHR], 0.80; 95% CI, 0.59-1.08; *P* = .15) (eFigure 4 in Supplement 1). The addition of cetuximab to radiotherapy did not significantly alter the LRF rate compared with radiotherapy alone (SHR, 1.26; 95% CI, 0.86-1.86; *P* = .23). After IPW, neither chemoradiation (0.62; 95% CI, 0.30-1.26; *P* = .19) nor cetuximab-based bioradiotherapy (SHR, 1.41; 95% CI, 0.86-2.29; *P* = .17) exhibited a significant difference regarding the incidence of LRFs compared with radiotherapy without concomitant treatment. Similar to the LRF analyses, there was no significant difference in the incidence of DMs after IPW between chemoradiation and radiotherapy (SHR, 0.70; 95% CI, 0.25-1.98; *P* = .50) or between cetuximab-based bioradiotherapy and radiotherapy (SHR, 1.13; 95% CI 0.58-2.19; *P* = .71) (eFigure 5 in Supplement 1).

### Subgroup Analysis

**Figure 3.**
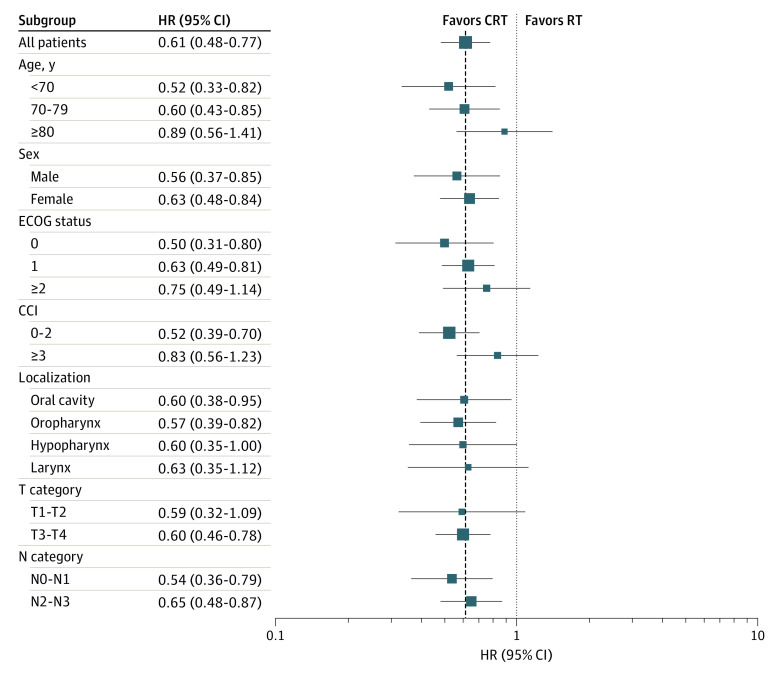
Subgroup Analyses Regarding the Association of Chemoradiation (CRT) With Overall Survival Among Older Patients (≥65 Years) With Head and Neck Squamous Cell Carcinoma Who Received Definitive Radiotherapy (RT) Between 2005 and 2019 CCI indicates Charlson Comorbidity Index; ECOG, Eastern Cooperative Oncology Group; HR, hazard ratio.

Chemoradiation was associated with a reduced hazard of death in most of the analyzed subgroups (Figure 3). The benefit was most pronounced in patients younger than 70 years (HR, 0.52; 95% C0.94I, 0.33-0.82) and patients between age 70 and 79 years (HR, 0.60; 95% CI, 0.43-0.85), while there was no significant difference between chemoradiation and radiotherapy among patients aged 80 years or older (HR, 0.89; 95% CI, 0.56-1.41). In the same manner, patients with ECOG status of 0 (HR, 0.50; 95% CI, 0.31-0.80) and 1 (HR, 0.63; 95% CI, 0.49-0.81) had the most pronounced benefit of chemoradiation, while there was no significant benefit for patients with ECOG status of 2 or higher (HR, 0.75; 95% CI, 0.49-1.14).

## Discussion

In this cohort study of 1044 older adults with LA-HNSCC, chemoradiation was associated with longer survival than radiotherapy alone, whereas the addition of cetuximab to radiotherapy was not associated with improved survival. The ideal treatment of older patients with LA-HNSCC is a matter of debate, considering the limited evidence due to underrepresentation or exclusion of older patients from landmark trials.^19^ The value of systemic treatment given concomitantly with definitive radiotherapy is a controversial topic, as there was no significant benefit of adding chemotherapy shown in the MACH-NC study.^3^ The increasing prevalence of frailty, comorbidities, and the higher vulnerability to chemotherapy-related toxic effects in older adults with HNSCCs may all contribute to the decreasing efficacy of chemoradiation in this population.^20,21^

Our analysis based on a multicenter registry with detailed clinical and treatment data from individual patients exhibits several advantages compared with the previous cancer database analyses. We included PFS as well as LRF and DM rate as further end points in addition to OS, and HPV status (for patients with oropharyngeal cancer) and smoking history were incorporated into the analysis.^8,9^ In comparison with the SEER analysis, we only analyzed the value of concomitant and not induction chemotherapy in our study, as induction chemotherapy generally does not improve survival in patients with LA-HNSCC undergoing chemoradiation.^3,22^ Furthermore, we only included HNSCCs of the oral cavity, oropharynx/hypopharynx, and larynx but not salivary glands, nasopharynx, or middle ear, as done in the SEER analysis.^8^ Therefore, our cohort is more homogeneous with respect to tumor localization, in which the general benefit of concomitant chemotherapy is more evident than, for instance, for salivary gland carcinomas.^23^

Similar to the results of the NCDB analysis, OS did not differ significantly between chemoradiation and radiotherapy within the subgroup of patients aged 80 years and older.^9^ The age breakpoint of 81 years in the NCDB analysis was not chosen by the authors but was the result of a recursive partitioning analysis. The authors hypothesized that the excess of toxic effects may have caused the absent benefit of chemoradiation in patients older than 80 years. A Taiwan Cancer Registry analysis of older patients with locally advanced oral cavity squamous cell carcinoma also reported on comparable OS between definitive chemoradiation and radiotherapy in the subgroup of the oldest cohort (>80 years).^24^ However, the evidence regarding the value of concomitant chemotherapy in very old patients with HNSCC is limited, and most analyses are derived from patients with oral cavity squamous cell carcinoma.^25,26,27^

The overall decreasing benefit of concomitant chemotherapy with higher patient age, as observed in the MACH-NC study, may be related to 2 factors: more (early) non–cancer-related deaths in older adults with HNSCCs that could mask improved locoregional control after chemoradiation,^28^ and increased chemotherapy-related toxic effects potentially increasing the risk of premature radiotherapy termination in the short-term^29,30^ and eventually even increasing the hazard of non–cancer-related deaths in the long-term.^31,32^ A potential explanation for the discrepant results between the MACH-NC and our study could be related to differences in the patient population (eg, probably higher proportion of patients with HPV-positive oropharyngeal carcinoma with no or limited alcohol and tobacco consumption in our cohort), improved supportive measures over time,^33^ more frequent use of chemotherapy regimens that are less toxic and easier to manage (eg, cisplatin weekly),^34,35,36^ and more modern radiotherapy treatment.^37^

Adding cetuximab to radiotherapy was not associated with longer OS or fewer LRFs compared with radiotherapy, according to our analysis. Three previous head-to-head comparisons between cisplatin-based chemoradiation and cetuximab-based bioradiotherapy for patients with HPV-positive oropharyngeal cancer noted inferior locoregional control rates in the respective cetuximab arms.^38,39,40^ Our data support previous findings from a SEER-based analysis in which cetuximab-based bioradiotherapy was found to be associated with comparable survival to radiotherapy alone, whereas chemoradiation was associated with superior survival in older patients with HNSCC after adjusting for patient- and tumor-related baseline variables.^41^

Multiple different chemotherapy regimens have been administered in the patient cohort analyzed in this study, reflecting the considerable heterogeneity between different treatment centers, especially given the lack of clear evidence or treatment recommendations in older adults with HNSCCs.^34,42^ Although the MACH-NC study did not support superiority of multiagent chemotherapy, and both US and European guidelines recommend the use of cisplatin in case of absent contraindications,^43,44^ there was a high proportion of older patients receiving multiagent chemotherapy in our cohort study. The higher prevalence of contraindications against cisplatin use in older patients (due to the higher prevalence of preexisting kidney disease or ototoxicity) may also have contributed to a higher use of multiagent protocols without cisplatin, such as mitomycin C plus fluorouracil, carboplatin plus paclitaxel, or carboplatin plus fluorouracil, all regimens with evidence from prospective trials in younger patients with HNSCC.^45,46,47,48^ However, because the medical decisions underlying the use of distinct chemotherapy protocols were not queried in our study, the proportion of patients ineligible to receive cisplatin therapy is unknown.

In the future, prospective trials in which the value of geriatric assessment and prognostic scores also can be examined are required to increase the scientific evidence regarding optimal treatment for older adults with HNSCC.^49,50,51^ Several studies, including phase 3 trials (novel agents such as NBTRX3 [NCT04892173] or hypofractionation with split-course [NCT01864850]) and prospective pilot studies (eg, hypofractionation [NCT04284540] or omitting coverage of elective sites of disease [NCT04832555]) are currently being conducted to improve nonsurgical treatment of older adults with HNSCC.

### Limitations

This study has limitations, albeit presenting what we believe to be the largest international multicenter cohort study of older patients with LA-HNSCC in which PFS, LRFs, and DMs based on comprehensive individual patient data were also analyzed. First, the SENIOR study is retrospective and therefore we aimed to control for the imbalances in the patient characteristics by using IPW. Although the chemoradiation group may still exhibit a favorable baseline characteristics profile after IPW due to variables that were not assessed in our study (eg, sarcopenia, frailty), all patients were considered to be fit enough to receive curative treatment. Second, we could not determine the cause of death in all patients, hampering calculations of other oncological end points, such as cancer-specific survival. Third, some variables, such as smoking and HPV status, were not available in a subset of patients, so we conducted k-nearest neighbor imputation for missing variables. Fourth, data about treatment breaks during radiotherapy courses were not available in our cohort. While the NCDB analysis observed a significantly longer overall treatment time in the chemoradiation than in the radiotherapy group among older adults with HNSCC, a large, Canadian single-center analysis could not observe differences in treatment interruption between younger and older patients with HNSCC undergoing radiotherapy or chemoradiation.^9,52^ Fifth, the absent significant benefit of chemoradiation with regard to the incidence of LRFs and DMs should be interpreted cautiously, as the number of events was relatively low (246 LRFs and 133 DMs) compared with the number of competing events (569 deaths), resulting in relatively large CIs in the competing risk analyses. The results of the LRF and DM analyses therefore should rather be interpreted as inconclusive than as evidence of no significant difference between chemoradiation and radiotherapy.

## Conclusions

In this cohort study of 1044 patients with LA-HNSCC aged 65 years and older, the addition of chemotherapy concomitantly to radiotherapy was associated with improved survival compared with radiotherapy alone, even after adjusting for potential confounding parameters. In contrast, patients who were treated with cetuximab-based bioradiotherapy had comparable outcomes to patients receiving definitive radiotherapy. Randomized prospective trials are needed to fully obtain the value of concomitant systemic treatment in the nonsurgical management of older adults with HNSCC.

## Notes

Supplement 1. **eTable 1.** Missing Data per Variable in the SENIOR Study Cohort **eTable 2.** Baseline Characteristics of Patients 65 Years and Older Who Underwent Definitive Radiotherapy for Locally Advanced Head and Neck Squamous Cell Carcinoma (2005-2019) **eTable 3.** Chemotherapy Regimens of the SENIOR Study Cohort **eTable 4.** Cox Proportional Hazard Regression Analysis (Unadjusted) for Overall Survival in Patients Aged 65 Years and Older Who Were Treated With Definitive Radiotherapy for Locally Advanced Head and Neck Squamous Cell Carcinoma (2005-2019) **eTable 5.** Cox Proportional Regression Analysis (Unadjusted) Regarding Progression-Free Survival in Patients Aged 65 Years and Older Who Were Treated With Definitive Radiotherapy for Locally Advanced Head and Neck Squamous Cell Carcinoma (2005-2019) **eTable 6.** Cox Proportional Regression Analysis (IPW-adjusted) Regarding Progression-Free Survival in Patients Aged 65 Years and Older Who Were Treated With Definitive Radiotherapy for Locally Advanced Head and Neck Squamous Cell Carcinoma (2005-2019) **eFigure 1.** Standardized Mean Differences Before and After Inverse Probability Weighting **eFigure 2.** Complete Case Analysis for Overall Survival of Older (≥65 Years) Head and Neck Squamous Cell Carcinoma Patients Receiving Radiotherapy Independent of Concomitant Systemic Treatment **eFigure 3.** Complete Case Analysis for Progression-Free Survival of Older (≥65 Years) Head and Neck Squamous Cell Carcinoma Patients Receiving Radiotherapy Independent of Concomitant Systemic Treatment **eFigure 4.** Cumulative Incidence of Locoregional Failures in Older (≥65 Years) Head and Neck Squamous Cell Carcinoma Patients Receiving Radiotherapy Depending on Concomitant Systemic Treatment **eFigure 5.** Cumulative Incidence of Distant Metastases in Older (≥65 Years) Head and Neck Squamous Cell Carcinoma Patients Receiving Radiotherapy Depending on Concomitant Systemic Treatment

Supplement 2.
Data Sharing Statement
