# Supplementary material for: Evaluation of Concomitant Systemic Treatment in Older Adults With Head and Neck Squamous Cell Carcinoma Undergoing Definitive Radiotherapy
Source: JAMA Netw Open. 2023 Feb 20;6(2):e230090. doi: 10.1001/jamanetworkopen.2023.0090 (PMC9941890; doi:10.1001/jamanetworkopen.2023.0090)
Supplement: Supplement 1. — eTable 1. Missing Data per Variable in the SENIOR Study Cohort eTable 2. Baseline Characteristics of Patients 65 Years and Older Who Underwent Definitive Radiotherapy for Locally Advanced Head and Neck Squamous Cell Carcinoma (2005-2019) eTable 3. Chemotherapy Regimens of the SENIOR Study Cohort eTable 4. Cox Proportional Hazard Regression Analysis (Unadjusted) for Overall Survival in Patients Aged 65 Years and Older Who Were Treated With Definitive Radiotherapy for Locally Advanced Head and Neck Squamous Cell Carcinoma (2005-2019) eTable 5. Cox Proportional Regression Analysis (Unadjusted) Regarding Progression-Free Survival in Patients Aged 65 Years and Older Who Were Treated With Definitive Radiotherapy for Locally Advanced Head and Neck Squamous Cell Carcinoma (2005-2019) eTable 6. Cox Proportional Regression Analysis (IPW-adjusted) Regarding Progression-Free Survival in Patients Aged 65 Years and Older Who Were Treated With Definitive Radiotherapy for Locally Advanced Head and Neck Squamous Cell Carcinoma (2005-2019) eFigure 1. Standardized Mean Differences Before and After Inverse Probability Weighting eFigure 2. Complete Case Analysis for Overall Survival of Older (≥65 Years) Head and Neck Squamous Cell Carcinoma Patients Receiving Radiotherapy Independent of Concomitant Systemic Treatment eFigure 3. Complete Case Analysis for Progression-Free Survival of Older (≥65 Years) Head and Neck Squamous Cell Carcinoma Patients Receiving Radiotherapy Independent of Concomitant Systemic Treatment eFigure 4. Cumulative Incidence of Locoregional Failures in Older (≥65 Years) Head and Neck Squamous Cell Carcinoma Patients Receiving Radiotherapy Depending on Concomitant Systemic Treatment eFigure 5. Cumulative Incidence of Distant Metastases in Older (≥65 Years) Head and Neck Squamous Cell Carcinoma Patients Receiving Radiotherapy Depending on Concomitant Systemic Treatment [file jamanetwopen-e230090-s001.pdf]

## Supplementary Online Content

Rühle A, Marschner S, Haderlein M, et al. Evaluation of concomitant systemic treatment in older adults with head and neck squamous cell carcinoma undergoing definitive radiotherapy. *JAMA Netw Open*. 2023;6(2):e230090. doi:10.1001/jamanetworkopen.2023.0090

**eTable 1.** Missing Data per Variable in the SENIOR Study Cohort

**eTable 2.** Baseline Characteristics of Patients 65 Years and Older Who Underwent Definitive Radiotherapy for Locally Advanced Head and Neck Squamous Cell Carcinoma (2005-2019)

**eTable 3.** Chemotherapy Regimens of the SENIOR Study Cohort

**eTable 4.** Cox Proportional Hazard Regression Analysis (Unadjusted) for Overall Survival in Patients Aged 65 Years and Older Who Were Treated With Definitive Radiotherapy for Locally Advanced Head and Neck Squamous Cell Carcinoma (2005-2019)

**eTable 5.** Cox Proportional Regression Analysis (Unadjusted) Regarding Progression-Free Survival in Patients Aged 65 Years and Older Who Were Treated With Definitive Radiotherapy for Locally Advanced Head and Neck Squamous Cell Carcinoma (2005-2019)

**eTable 6.** Cox Proportional Regression Analysis (IPW-adjusted) Regarding Progression-Free Survival in Patients Aged 65 Years and Older Who Were Treated With Definitive Radiotherapy for Locally Advanced Head and Neck Squamous Cell Carcinoma (2005-2019)

**eFigure 1.** Standardized Mean Differences Before and After Inverse Probability Weighting

**eFigure 2.** Complete Case Analysis for Overall Survival of Older (≥65 Years) Head and Neck Squamous Cell Carcinoma Patients Receiving Radiotherapy Independent of Concomitant Systemic Treatment

**eFigure 3.** Complete Case Analysis for Progression-Free Survival of Older (≥65 Years) Head and Neck Squamous Cell Carcinoma Patients Receiving Radiotherapy Independent of Concomitant Systemic Treatment

**eFigure 4.** Cumulative Incidence of Locoregional Failures in Older (≥65 Years) Head and Neck Squamous Cell Carcinoma Patients Receiving Radiotherapy Depending on Concomitant Systemic Treatment

**eFigure 5.** Cumulative Incidence of Distant Metastases in Older (≥65 Years) Head and Neck Squamous Cell Carcinoma Patients Receiving Radiotherapy Depending on Concomitant Systemic Treatment

This supplementary material has been provided by the authors to give readers additional information about their work.

© 2023 Rühle A et al. *JAMA Network Open.*

## eTable 1. Missing Data per Variable in the SENIOR Study Cohort.

| **Characteristic** | **Patients with missing data** |
|---|---|
| Age | 0 |
| Sex | 0 |
| ECOG | 21 |
| CCI | 2 |
| Smoking | 166 |
| Localization | 0 |
| Clinical T stage | 0 |
| Clinical N stage | 1 |
| HPV status of oropharynx carcinomas | 219 |
| Radiotherapy dose | 0 |
| Radiotherapy completion | 0 |
| Concomitant systemic treatment | 0 |

© 2023 Rühle A et al. *JAMA Network Open.*

**eTable 2. Baseline Characteristics of Patients 65 Years and Older Who Underwent Definitive Radiotherapy for Locally Advanced Head and Neck Squamous Cell Carcinoma (2005-2019).** CCI indicates Charlson Comorbidity Index; Eastern Cooperative Oncology Group; HPV, human papillomavirus.

| Characteristic | No. (%) |
|---|---|
| **Age, median (IQR), y** | 73 (69-78) |
| **Sex** | |
| Female | 310 (29.7) |
| Male | 734 (70.3) |
| **ECOG**[a] | |
| 0 | 269 (26.3) |
| 1 | 530 (51.8) |
| ≥2 | 224 (21.9) |
| **CCI, median (IQR)**[b] | 2 (0-3) |
| **Smoking**[c] | |
| Never smoker/limited smoking | 306 (34.9) |
| Smoking >10 pack-years | 572 (65.1) |
| **Localization** | |
| Oral cavity | 160 (15.3) |
| Oropharynx | 523 (50.1) |
| Hypopharynx | 171 (16.4) |
| Larynx | 152 (14.6) |
| Oro-/Hypopharynx | 38 (3.6) |
| **Clinical T stage** | |
| cT1 | 46 (4.4) |
| cT2 | 135 (12.9) |
| cT3 | 344 (33.0) |
| cT4 | 519 (49.7) |
| **Clinical N stage**[d] | |
| cN0 | 196 (18.8) |
| cN1 | 136 (13.0) |
| cN2a | 24 (2.3) |
| cN2b | 234 (22.4) |
| cN2c | 184 (17.6) |
| cN2, not specified | 203 (19.5) |
| cN3 | 64 (6.1) |
| **HPV status of oropharynx carcinomas**[e] | |
| HPV-positive | 186 (61.2) |
| HPV-negative | 118 (38.8) |
| **Radiotherapy dose, median (IQR), Gy** | 70.0 (69.3-70.0) |
| **Radiotherapy completion** | |
| Radiotherapy completed | 933 (89.4) |
| Radiotherapy not completed | 111 (10.6) |
| **Concomitant systemic treatment** | |
| Chemotherapy | 677 (64.8) |
| Cetuximab | 133 (12.7) |
| No systemic treatment | 234 (22.4) |

[a] 1023 patients
[b] 1042 patients
[c] 878 patients
[d] 1043 patients
[e] 304 patients

© 2023 Rühle A et al. *JAMA Network Open.*

## eTable 3. Chemotherapy Regimens of the SENIOR Study Cohort.

| **Chemotherapy** | **Number** |
|---|---|
| Cisplatin | 298 |
| Cisplatin + 5-fluorouracil | 137 |
| Carboplatin | 68 |
| Mitomycin C + 5-fluorouracil | 64 |
| Mitomycin C | 50 |
| Carboplatin + paclitaxel | 14 |
| Cisplatin + paclitaxel | 13 |
| Paclitaxel | 12 |
| Others | 21 |

© 2023 Rühle A et al. *JAMA Network Open.*

**eTable 4. Cox Proportional Hazard Regression Analysis (Unadjusted) for Overall Survival in Patients Aged 65 Years and Older Who Were Treated With Definitive Radiotherapy for Locally Advanced Head and Neck Squamous Cell Carcinoma (2005-2019).** CCI indicates Charlson Comorbidity Index; ECOG, Eastern Cooperative Oncology Group; HPV, human papillomavirus.

| **Radiotherapy versus chemoradiation** | | | **Radiotherapy versus radiotherapy plus cetuximab** | | |
|---|---|---|---|---|---|
| **Characteristic** | **HR (95% CI)** | ***p*** | **Characteristic** | **HR [95% CI]** | ***p*** |
| **Age** | 1.03 (1.01-1.04) | .003 | **Age** | 1.02 (1.00-1.04) | .06 |
| **Sex (reference, female)** | 1.31 (1.07-1.61) | .009 | **Sex (reference, female)** | 0.93 (0.68-1.27) | .66 |
| **ECOG status (reference, 0)** | | | **ECOG status (reference, 0)** | | |
| **1** | 1.62 (1.26-2.07) | <.001 | **1** | 1.33 (0.87-2.05) | .19 |
| **2** | 1.99 (1.47-2.69) | <.001 | **2** | 1.76 (1.10-2.84) | .02 |
| **3** | 1.72 (0.94-3.12) | .08 | **3** | 2.31 (1.14-4.67) | .02 |
| **CCI** | 1.07 (1.02-1.12) | .01 | **CCI** | 1.04 (0.96-1.12) | 0.34 |
| **Smoking (reference, never/limited smoking)** | 1.16 (0.94-1.43) | .17 | **Smoking (reference, never/limited smoking)** | 1.58 (1.17-2.14) | .003 |
| **Localization (reference, oral cavity)** | | | **Localization (reference, oral cavity)** | | |
| **Oropharynx** | 0.60 (0.46-0.77) | <.001 | **Oropharynx** | 0.47 (0.32-0.69) | <.001 |
| **Hypopharynx** | 0.67 (0.50-0.90) | .007 | **Hypopharynx** | 0.79 (0.51-1.23) | .31 |
| **Larynx** | 0.54 (0.39-0.74) | <.001 | **Larynx** | 0.48 (0.31-0.76) | .002 |
| **Oro-/Hypopharynx (multi-level)** | 0.73 (0.42-1.29) | .28 | **Oro-/Hypopharynx (multi-level)** | 0.42 (0.10-1.77) | .24 |
| **Clinical T stage (reference, cT1)** | | | **Clinical T stage (reference, cT1)** | | |
| **T2** | 0.88 (0.51-1.51) | .65 | **T2** | 1.39 (0.60-3.25) | .44 |
| **T3** | 1.27 (0.78-2.09) | .33 | **T3** | 2.11 (0.95-4.68) | .07 |
| **T4** | 1.54 (0.95-2.48) | .08 | **T4** | 2.21 (1.00-4.86) | .05 |
| **Clinical N stage (reference, cN0)** | | | **Clinical N stage (reference, cN0)** | | |
| **cN1** | 1.22 (0.86-1.73) | .26 | **cN1** | 0.75 (0.46-1.22) | .24 |
| **cN2** | 1.52 (1.18-1.96) | .001 | **cN2** | 1.18 (0.84-1.67) | .34 |
| **cN3** | 2.35 (1.52-3.63) | <.001 | **cN3** | 1.54 (0.85-2.78) | .15 |
| **HPV status (reference, HPV-positive)** | 2.48 (1.74-3.53) | <.001 | **HPV status (reference, HPV-positive)** | 1.72 (1.09-2.72) | .02 |
| **Concomitant chemotherapy** | 0.62 (0.49-0.78) | <.001 | **Concomitant cetuximab** | 0.90 (0.67-1.22) | .51 |

**eTable 5. Cox Proportional Regression Analysis (Unadjusted) Regarding Progression-Free Survival in Patients Aged 65 Years and Older Who Were Treated With Definitive Radiotherapy for Locally Advanced Head and Neck Squamous Cell Carcinoma (2005-2019).** CCI indicates Charlson Comorbidity Index; ECOG, Eastern Cooperative Oncology Group; HPV, human papillomavirus.

| **Radiotherapy versus chemoradiation** | | | **Radiotherapy versus radiotherapy plus cetuximab** | | |
|---|---|---|---|---|---|
| **Characteristic** | **HR (95% CI)** | ***p*** | **Characteristic** | **HR [95% CI]** | ***P*** |
| **Age** | 1.02 (1.01, 1.04) | .006 | **Age** | 1.02 (1.00, 1.04) | .06 |
| **Sex (reference, female)** | 1.26 (1.05, 1.52) | .01 | **Sex (reference, female)** | 1.09 (0.81, 1.46) | .58 |
| **ECOG status (reference, 0)** | | | **ECOG status (reference, 0)** | | |
| **1** | 1.59 (1.27, 1.99) | .001 | **1** | 1.14 (0.77, 1.68) | .51 |
| **2** | 1.95 (1.48, 2.58) | .001 | **2** | 1.51 (0.98, 2.32) | .06 |
| **3** | 1.67 (0.94, 2.97) | .08 | **3** | 2.07 (1.04, 4.10) | .04 |
| **CCI** | 1.03 (0.99, 1.08) | .17 | **CCI** | 0.99 (0.92, 1.07) | .82 |
| **Smoking (reference, never/limited smoking)** | 1.17 (0.96, 1.42) | .12 | **Smoking (reference, never/limited smoking)** | 1.46 (1.10, 1.94) | .01 |
| **Localization (reference, oral cavity)** | | | **Localization (reference, oral cavity)** | | |
| **Oropharynx** | 0.55 (0.43, 0.70) | .001 | **Oropharynx** | 0.48 (0.33, 0.70) | <.001 |
| **Hypopharynx** | 0.69 (0.53, 0.91) | .009 | **Hypopharynx** | 0.76 (0.50, 1.17) | .22 |
| **Larynx** | 0.55 (0.40, 0.74) | .001 | **Larynx** | 0.55 (0.36, 0.84) | .006 |
| **Oro-/Hypopharynx (multi-level)** | 0.68 (0.41, 1.14) | .14 | **Oro-/Hypopharynx (multi-level)** | 0.84 (0.33, 2.15) | .71 |
| **Clinical T stage (reference, cT1)** | | | **Clinical T stage (reference, cT1)** | | |
| **cT2** | 0.91 (0.56, 1.49) | .70 | **cT2** | 1.40 (0.63, 3.09) | .40 |
| **cT3** | 1.27 (0.81, 1.99) | .30 | **cT3** | 2.22 (1.06, 4.67) | .04 |
| **cT4** | 1.49 (0.96, 2.31) | .08 | **cT4** | 2.47 (1.18, 5.15) | .02 |
| **Clinical N stage (reference, cN0)** | | | **Clinical N stage (reference, cN0)** | | |
| **cN1** | 1.20 (0.87, 1.65) | .26 | **cN1** | 0.91 (0.58, 1.43) | .69 |
| **cN2** | 1.40 (1.11, 1.77) | .005 | **cN2** | 1.23 (0.88, 1.71) | .22 |
| **cN3** | 2.34 (1.58, 3.47) | <.001 | **cN3** | 2.35 (1.39, 3.96) | .001 |
| **HPV status (reference, HPV-positive)** | 1.75 (1.30, 2.37) | <.001 | **HPV status (reference, HPV-positive)** | 1.41 (0.93, 2.15) | .11 |
| **Concomitant chemotherapy** | 0.69 (0.56, 0.85) | <.001 | **Concomitant cetuximab** | 0.99 (0.75, 1.31) | .97 |

© 2023 Rühle A et al. *JAMA Network Open.*

**eTable 6. Cox Proportional Regression Analysis (IPW-adjusted) Regarding Progression-Free Survival in Patients Aged 65 Years and Older Who Were Treated With Definitive Radiotherapy for Locally Advanced Head and Neck Squamous Cell Carcinoma (2005-2019).** CCI indicates Charlson Comorbidity Index; ECOG, Eastern Cooperative Oncology Group; HPV, human papillomavirus.

| Radiotherapy versus chemoradiation | | | Radiotherapy versus radiotherapy plus cetuximab | | |
|---|---|---|---|---|---|
| **Characteristic** | **HR (95% CI)** | ***p*** | **Characteristic** | **HR [95% CI]** | ***P*** |
| **Age** | 1.02 (1.01-1.04) | .01 | **Age** | 1.02 (1.00-1.04) | .08 |
| **Sex (reference, female)** | 1.27 (1.02-1.57) | .03 | **Sex (reference, female)** | 1.09 (0.79-1.50) | .60 |
| **ECOG status (reference, 0)** | | | **ECOG status (reference, 0)** | | |
| **1** | 1.55 (1.21-1.97) | <.001 | **1** | 1.13 (0.77-1.67) | .53 |
| **2** | 1.85 (1.35-2.54) | <.001 | **2** | 1.54 (0.99-2.41) | .06 |
| **3** | 1.62 (0.74-3.54) | .22 | **3** | 2.14 (1.10-4.15) | .02 |
| **CCI** | 1.02 (0.97-1.07) | .46 | **CCI** | 0.98 (0.91-1.06) | .69 |
| **Smoking (reference, never/limited smoking)** | 1.25 (0.99-1.57) | .06 | **Smoking (reference, never/limited smoking)** | 1.47 (1.10-1.97) | .009 |
| **Localization (reference, oral cavity)** | | | **Localization (reference, oral cavity)** | | |
| **Oropharynx** | 0.53 (0.41-0.70) | <.001 | **Oropharynx** | 0.45 (0.30-0.69) | <.001 |
| **Hypopharynx** | 0.66 (0.49-0.88) | .004 | **Hypopharynx** | 0.72 (0.44-1.16) | 0.18 |
| **Larynx** | 0.54 (0.38-0.76) | <.001 | **Larynx** | 0.55 (0.35-0.86) | .009 |
| **Oro-/Hypopharynx (multi-level)** | 0.69 (0.44-1.08) | 0.11 | **Oro-/Hypopharynx (multi-level)** | 0.83 (0.40-1.74) | .62 |
| **Clinical T stage (reference, cT1)** | | | **Clinical T stage (reference, cT1)** | | |
| **cT2** | 1.04 (0.65-1.67) | .85 | **cT2** | 1.75 (0.82-3.72) | .14 |
| **cT3** | 1.20 (0.79-1.83) | .40 | **cT3** | 2.43 (1.21-4.87) | .01 |
| **cT4** | 1.49 (0.99-2.25) | .06 | **cT4** | 2.84 (1.42-5.67) | .003 |
| **Clinical N stage (reference, cN0)** | | | **Clinical N stage (reference, cN0)** | | |
| **cN1** | 1.11 (0.78-1.59) | .57 | **cN1** | 0.93 (0.60-1.43) | .73 |
| **cN2** | 1.49 (1.14-1.96) | .004 | **cN2** | 1.30 (0.90-1.87) | .16 |
| **cN3** | 2.10 (1.40-3.14) | <.001 | **cN3** | 2.25 (1.33-3.81) | .003 |
| **HPV status (reference, HPV-positive)** | 1.69 (1.19-2.38) | .003 | **HPV status (reference, HPV-positive)** | 1.27 (0.82-1.96) | .29 |
| **Concomitant chemotherapy** | 0.64 (0.51-0.80) | <.001 | **Concomitant cetuximab** | 1.00 (0.74-1.34) | .98 |

© 2023 Rühle A et al. *JAMA Network Open.*

**eFigure 1. Standardized Mean Differences Before and After Inverse Probability Weighting.** CCI, Charlson Comorbidity Index; CRT, chemoradiation; ECOG, Eastern Cooperative Oncology Group; HPV, human papillomavirus; IPW, inverse probability weighting; RT, radiotherapy; SMD, standardized mean difference.

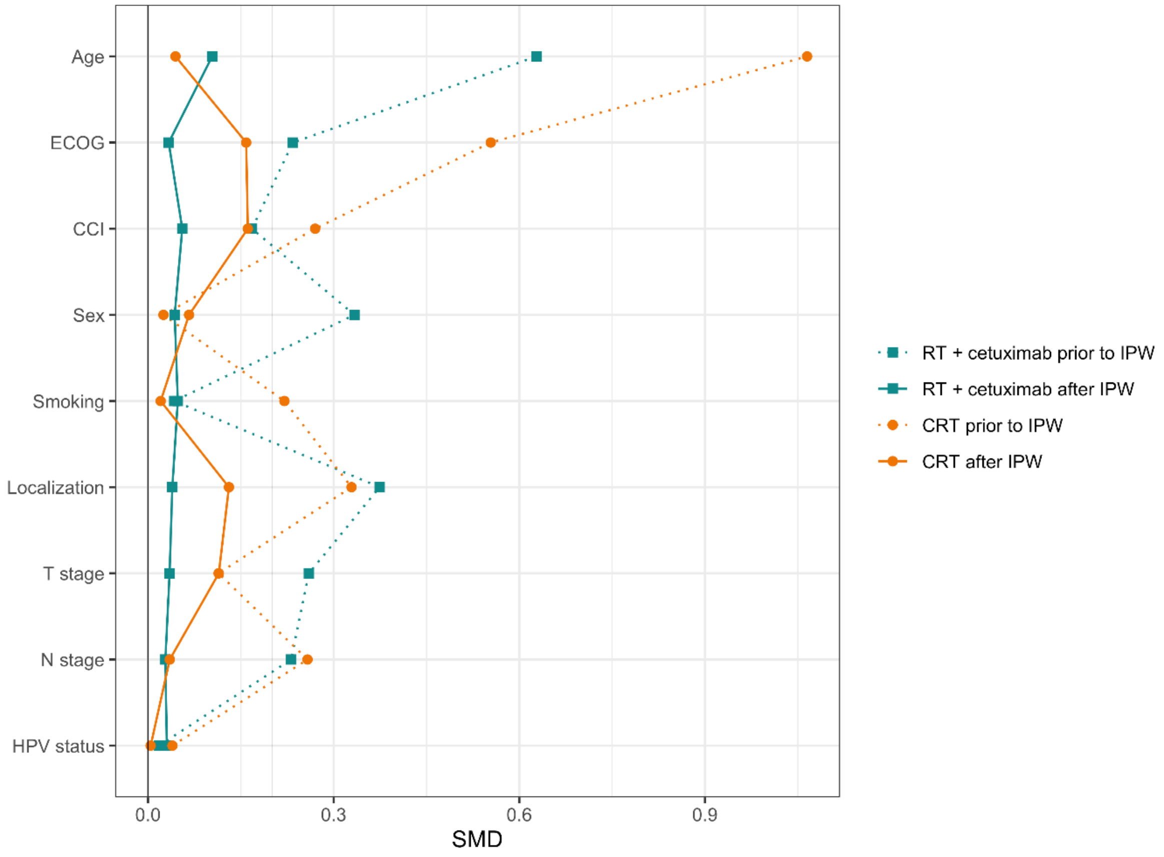


© 2023 Rühle A et al. *JAMA Network Open.*

**eFigure 2. Complete case analysis for Overall Survival of Older (≥65 Years) Head and Neck Squamous Cell Carcinoma Patients Receiving Radiotherapy Independent of Concomitant Systemic Treatment.** Only patients without missing variables (age, sex, ECOG, comorbidities, smoking, tumor localization, T stage, N stage and HPV status) were included. CRT indicates chemoradiation; HR, hazard ratio; RT, radiotherapy. Inverse Probability Weighting (IPW) was used to balance the groups regarding the baseline covariates.

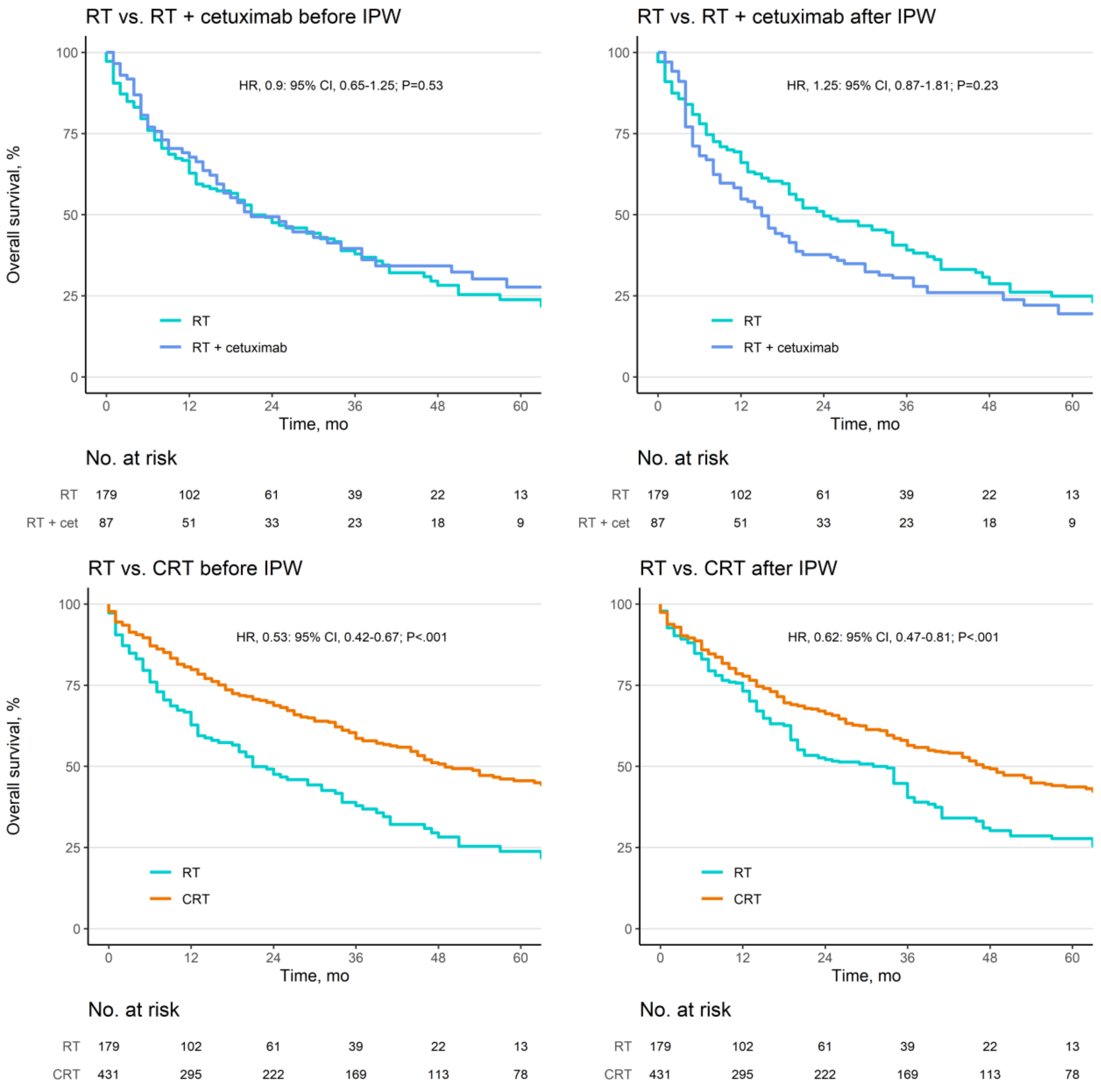


© 2023 Rühle A et al. *JAMA Network Open.*

**eFigure 3. Complete case analysis for Progression-free Survival of Older (≥65 Years) Head and Neck Squamous Cell Carcinoma Patients Receiving Radiotherapy Independent of Concomitant Systemic Treatment.** Only patients without missing variables (age, sex, ECOG, comorbidities, smoking, tumor localization, T stage, N stage and HPV status) were included. CRT indicates chemoradiation; HR, hazard ratio; RT, radiotherapy. Inverse Probability Weighting (IPW) was used to balance the groups regarding the baseline covariates.

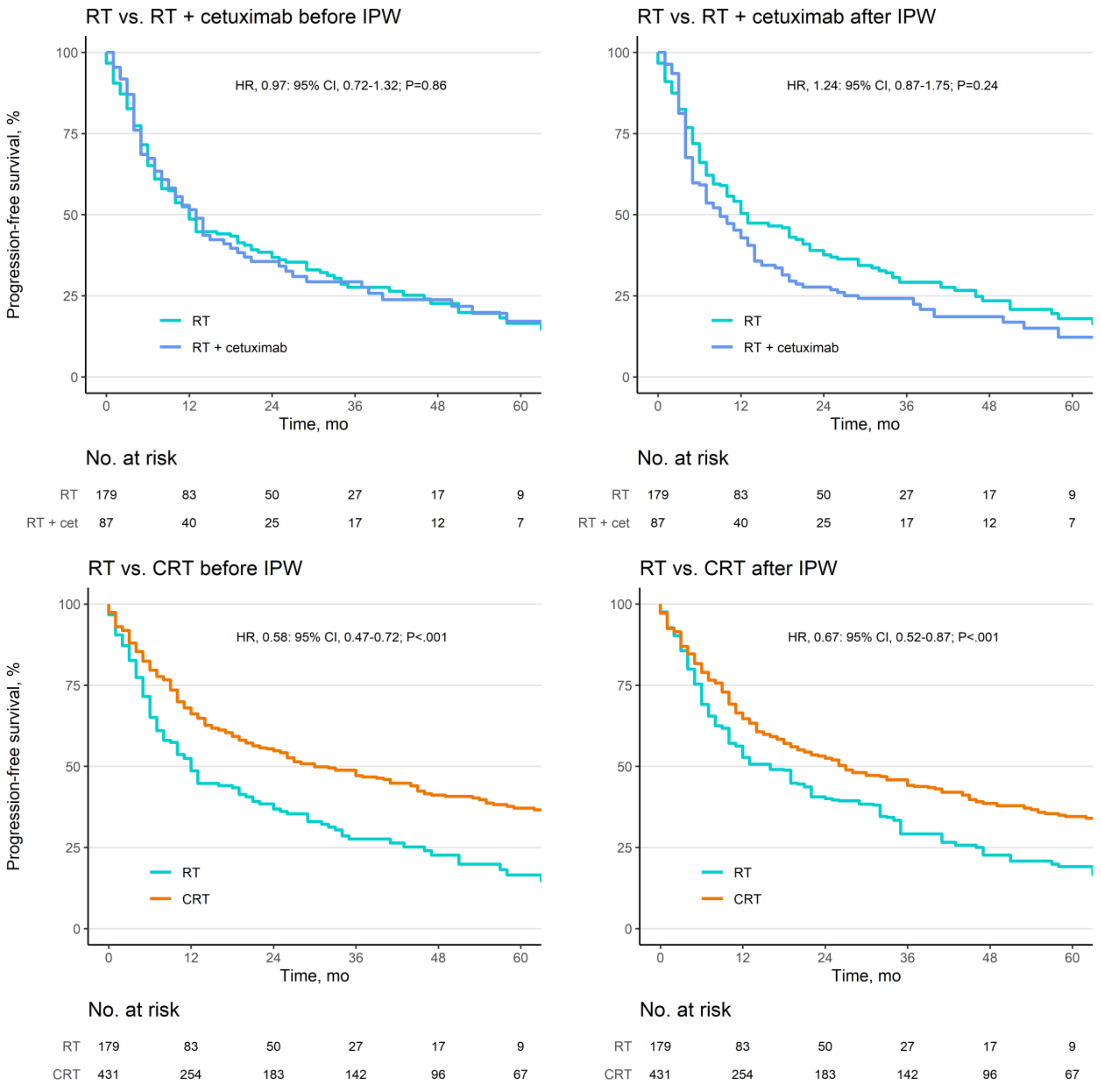


© 2023 Rühle A et al. *JAMA Network Open.*

**eFigure 4. Cumulative Incidence of Locoregional Failures in Older (≥65 Years) Head and Neck Squamous Cell Carcinoma Patients Receiving Radiotherapy Depending on Concomitant Systemic Treatment.** cet, cetuximab; CRT, chemoradiation; IPW, inverse probability weighting; LRFs, locoregional failures; RT, radiotherapy. SHR, subdistribution hazard ratio. Note that the number of patients in the RT group is lower (232 instead of 234), as two patients with ECOG=4 could not be matched using IPW.

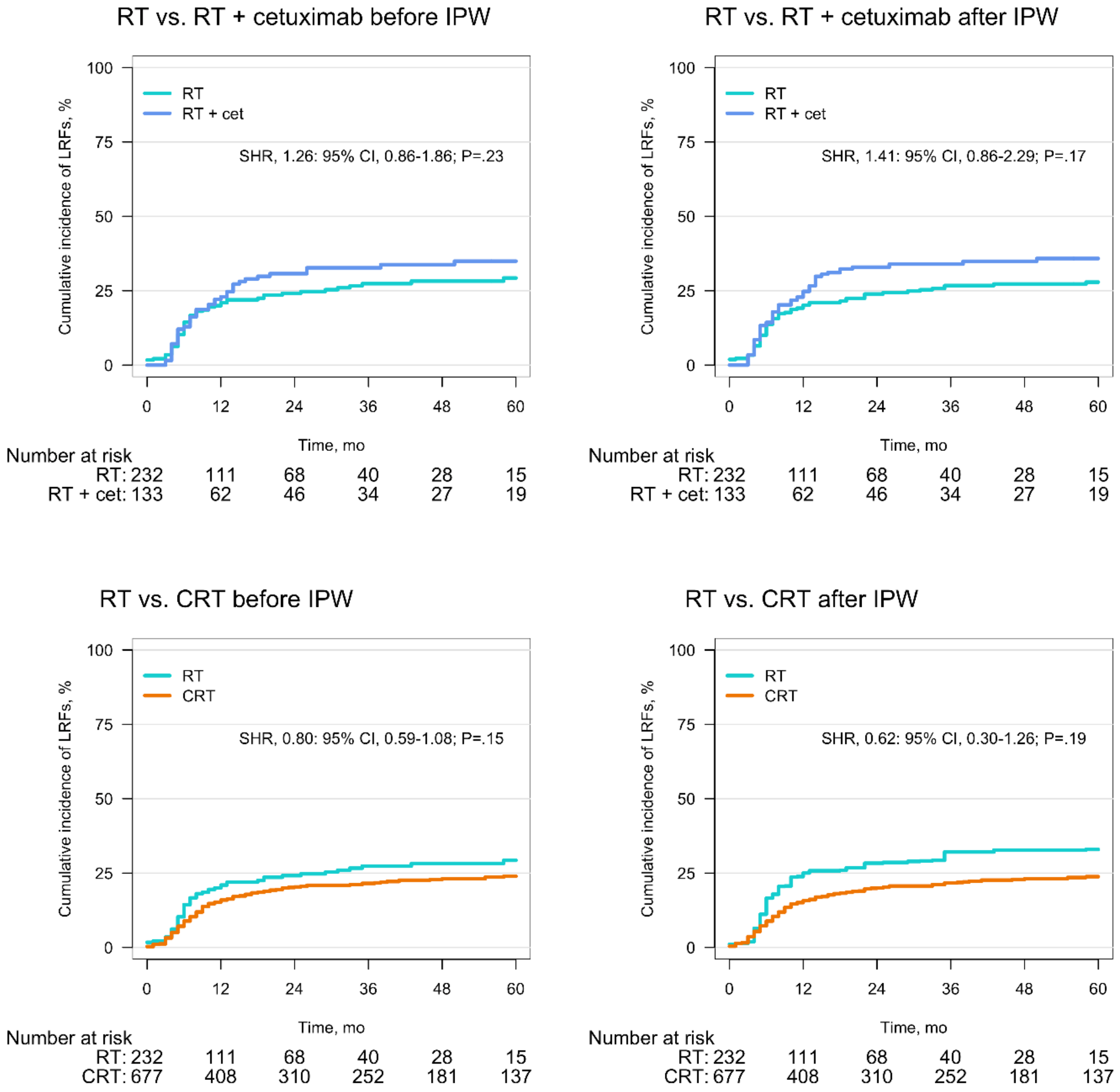


© 2023 Rühle A et al. *JAMA Network Open.*

**eFigure 5. Cumulative Incidence of Distant Metastases in Older (≥65 Years) Head and Neck Squamous Cell Carcinoma Patients Receiving Radiotherapy Depending on Concomitant Systemic Treatment.** cet, cetuximab; CRT, chemoradiation; IPW, inverse probability weighting; DMs, distant metastases; RT, radiotherapy. SHR, subdistribution hazard ratio. Note that the number of patients in the RT group is lower (232 instead of 234), as two patients with ECOG=4 could not be matched using IPW.

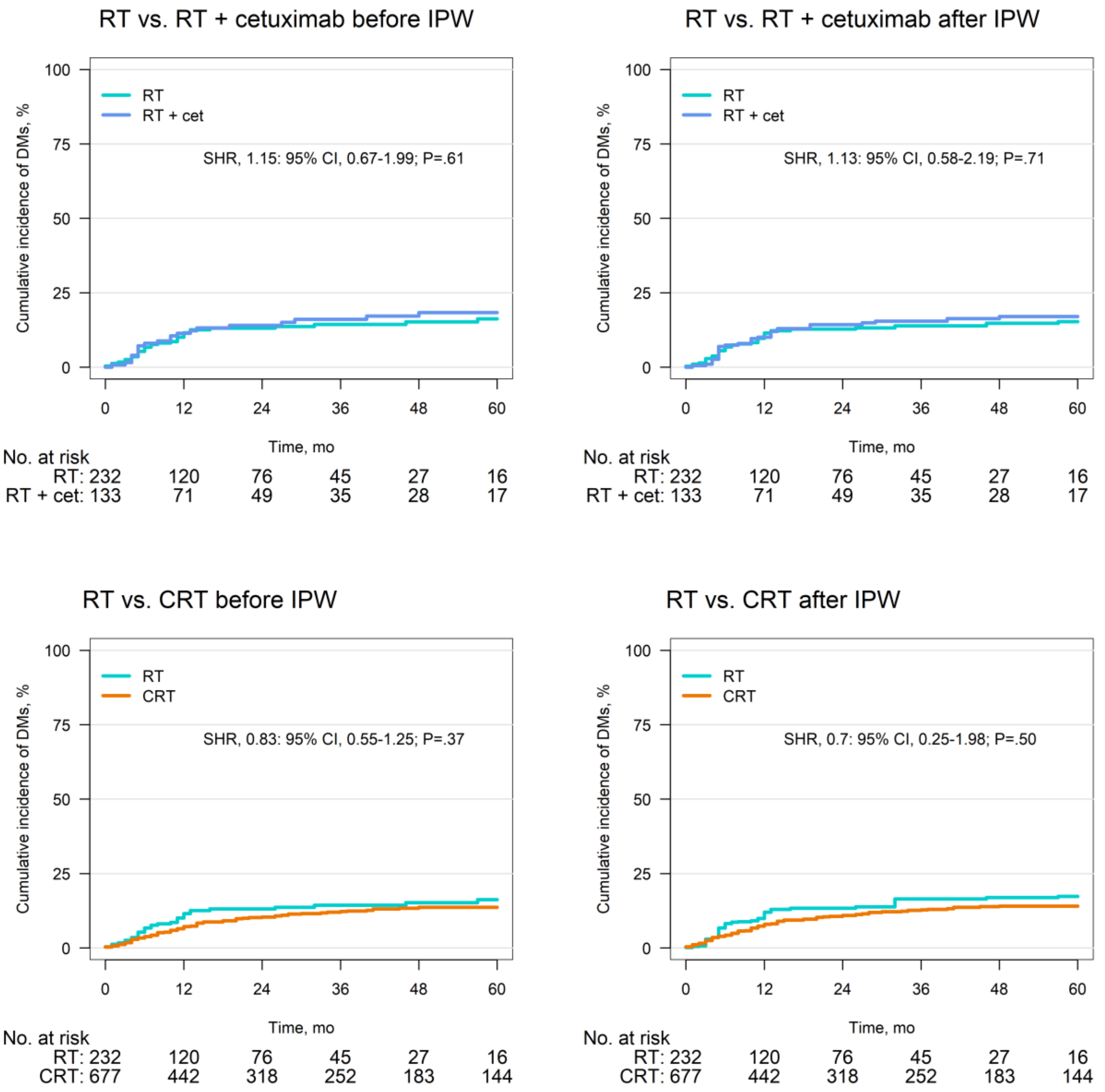


© 2023 Rühle A et al. *JAMA Network Open.*
