# Supplementary material for: Evaluation of Concomitant Systemic Treatment in Older Adults With Head and Neck Squamous Cell Carcinoma Undergoing Definitive Radiotherapy
Source: JAMA Netw Open. 2023 Feb 20;6(2):e230090. doi: 10.1001/jamanetworkopen.2023.0090 (PMC9941890; doi:10.1001/jamanetworkopen.2023.0090)
Supplement: Supplement 2. — Data Sharing Statement [file jamanetwopen-e230090-s002.pdf]

# Data Sharing Statement

Rühle. Evaluation of Concomitant Systemic Treatment in Older Adults With Head and Neck Squamous Cell Carcinoma Undergoing Definitive Radiotherapy. *JAMA Netw Open*. Published February 20, 2023. doi:10.1001/jamanetworkopen.2023.0090

**Data**
**Data available:** No

**Additional Information**
**Explanation for why data not available:** National Data Protection Regulations.
